# LONGING FOR LOVE: FINANCIAL STRAIN AND LATER LIFE MENTAL HEALTH IN THE US

**DOI:** 10.1093/geroni/igac059.1932

**Published:** 2022-12-20

**Authors:** Tirth Bhatta, Jeffrey Albert, Stacy Torres, Eva Kahana, Nirmala Lekhak, Timothy Goler

**Affiliations:** University of Nevada Las Vegas, Las Vegas, Nevada, United States; Case Western Reserve University, Cleveland, Ohio, United States; University of California, San Francisco, San Francisco, California, United States; Case Western Reserve University, Cleveland, Ohio, United States; University of Nevada, Las Vegas, Nevada, United States; Norfolk State University, Norfolk, Virginia, United States

## Abstract

**Objectives:**

Emotional support has been consistently identified as a mechanism through which socioeconomic resources influence mental health outcomes. Despite emerging evidence that compassionate love has a beneficial effect on mental health, its distribution across levels of financial strain and subsequent role in mediating the effect of financial strain on later life mental health have yet to be examined.

**Methods:**

Based on our nationwide web-based survey of adults aged 50 years and older (n=1751), we conducted a mediation analysis to estimate the direct and indirect effects (via two mediators, feeling loved and emotional support) of financial strain on depressive symptoms and anxiety.

**Results:**

We documented a statistically significant overall effect of financial strain on depressive symptoms (b=0.14, p-value< 0.001) and anxiety (b=0.196, p-value< 0.001). We found a statistically significant path-specific effect (from financial hardship) through compassionate love alone on depressive symptoms (b=0.018, p-value=0.003) and anxiety (b=0.016, p-value=0.005), but did not find effects for paths through emotional support. There was also a significant direct effect of financial strain on both depressive symptoms and anxiety. Discussion: Our study advances a new line of research by looking at the role of compassionate love in transmitting the effects of financial strain on mental health in later life. Findings suggest that the detrimental effect of financial hardship on mental health operates through its negative effect on the receipt of compassionate love, rather than by its impact on emotional support.

